# TMEM106B C‐terminal fragments drive a distinct neurodegenerative proteinopathy in C*. elegans*.

**DOI:** 10.1002/alz.088591

**Published:** 2025-01-03

**Authors:** Brian C. Kraemer, Ruben Riordan

**Affiliations:** ^1^ University of Washington School of Medicine, Seattle, WA USA; ^2^ GRECC VA Puget Sound, Seattle, WA USA; ^3^ University of Washington, Seattle, WA USA

## Abstract

**Background:**

Genetic variation of lysosomal protein, transmembrane protein 106B (TMEM106B) has long been known as a risk factor for a diverse range of neurodegenerative disorders, especially FTLD with progranulin (GRN) haplo‐insufficiency, though the mechanisms involved are not yet understood. Recently, through advances in cryo‐electron microscopy (cryo‐EM), aggregates of the C‐Terminal domain of TMEM106B (TMEM CT) were shown to make up previously unidentifiable protein aggregates in the brains of human FTLD, AD, progressive supranuclear palsy (PSP), and dementia with Lewy Bodies (DLB) patients.

**Methods:**

To determine the TMEM CT aggregation propensity and neurodegenerative potential, we generated a new transgenic C. *elegans* proteinopathy model pan‐neuronally expressing the TMEM CT fragment constituting the fibrillar core in FTLD‐TDP cases.

**Results:**

We found that C. *elegans* pan‐neuronal expression of TMEM CT causes neuronal dysfunction as evidenced by behavioral analysis. TMEM c‐terminal fragments accumulate as detergent insoluble material. Behavioral dysfunction was accompanied by neurodegeneration as illustrated by loss of GABAergic neurons. To investigate the mechanisms driving TMEM106B proteinopathy, we aimed to explore the impact of GRN loss on the neurodegenerative effect of TMEM CT expression. To this end, we generated TMEM CT expressing C. *elegans* with PRGN loss of function. We found that loss of PGRN did not alter TMEM phenotypes confirming PGRN loss of function is upstream of TMEM aggregation. We also observed TMEM neurodegenerative phenotypes exhibit a distinct set of genetic modifiers as strong suppressors of tauopathy such as spop‐1, sut‐2 sut‐6 show only modest modification of TMEM proteinopathy.

**Conclusions:**

Taken together, our data suggest expression of TMEM CT in C. *elegans* neurons provides a useful model for the functional characterization of TMEM106B proteinopathy in neurodegenerative disease. Comparison with other proteinopathy models will inform potential disease mechanisms.

